# Aloperine executes antitumor effects against multiple myeloma through dual apoptotic mechanisms

**DOI:** 10.1186/s13045-015-0120-x

**Published:** 2015-03-15

**Authors:** He Wang, Shu Yang, Hong Zhou, Mingna Sun, Lingran Du, Minyan Wei, Meixia Luo, Jingzhu Huang, Hongzhu Deng, Yinghong Feng, Jun Huang, Yi Zhou

**Affiliations:** Department of Oncology, The Second Affiliated Hospital of Guangzhou Medical University, Guangzhou, Guangdong 510260 China; College of Pharmaceutics Science, Guangzhou Medical University, Guangzhou, Guangdong 510182 China; Department of Oncology, The First Affiliated Hospital of Guangdong Pharmaceutical University, Guangzhou, Guangdong 510080 China; The Second Affiliated Hospital of Guangzhou University of Traditional Chinese Medicine, Guangzhou, Guangdong 510521 China; School of the Traditional Chinese Medicine, Southern Medical University, Guangzhou, Guangdong 510515 China; College of Basic Medicine, Guangzhou Medical University, Guangzhou, Guangdong 510182 China

**Keywords:** Aloperine, cFLIP, PTEN, Multiple myeloma

## Abstract

**Background:**

Aloperine, a natural alkaloid constituent isolated from the herb *Sophora alopecuroides* displays anti-inflammatory properties *in vitro* and *in vivo*. Our group previously demonstrated that aloperine significantly induced apoptosis in colon cancer SW480 and HCT116 cells. However, its specific target(s) remain to be discovered in multiple myeloma (MM) and have not been investigated.

**Methods:**

Human myeloma cell lines (*n* = 8), primary myeloma cells (*n* = 12), drug-resistant myeloma cell lines (*n* = 2), and animal models were tested for their sensitivity to aloperine in terms of proliferation and apoptosis both *in vitro* and *in vivo*, respectively. We also examined the functional mechanisms underlying the apoptotic pathways triggered by aloperine.

**Results:**

Aloperine induced MM cell death in a dose- and time-dependent manner, even in the presence of the proliferative cytokines interleukin-6 and insulin-like growth factor I. Mechanistic studies revealed that aloperine not only activated caspase-8 and reduced the expression of FADD-like interleukin-1β-converting enzyme (FLICE)*-*like inhibitory protein long (FLIPL) and FLICE-inhibitory proteins (FLIPS) but also activated caspase-9 and decreased the expression of phosphorylated (p)-PTEN. Moreover, co-activation of the caspase-8/cellular FLICE-inhibitory protein (cFLIP)- and caspase-9/p-PTEN/p-AKT-dependent apoptotic pathways by aloperine caused irreversible inhibition of clonogenic survival. Aloperine induce more MM apoptosis with tumor necrosis factor-related apoptosis-inducing ligand (TRAIL) or borterzomib. A U266 xenograft tumor model and 5T33 MM cells recapitulated the antitumor efficacy of aloperine, and the animals displayed excellent tolerance of the drug and few adverse effects.

**Conclusions:**

Aloperine has multifaceted antitumor effects on MM cells. Our data support the clinical development of aloperine for MM therapy.

**Electronic supplementary material:**

The online version of this article (doi:10.1186/s13045-015-0120-x) contains supplementary material, which is available to authorized users.

## Introduction

Multiple myeloma (MM) is a clonal malignancy of plasma cells that is characterized by the presence of a monoclonal protein in the serum and/or urine, widespread osteolysis, renal failure, and anemia [1,2]. MM survival rates, which have historically ranged from 3 to 5 years, can now exceed 10 years due to the advent of high-dose therapy with autologous stem cell transplantation in combination with novel chemotherapeutic agents [3,4]. However, high-dose chemotherapy produces drug resistance in MM patients, illustrating the urgent need for more effective treatments.

Current treatment approaches aim to induce apoptosis, a form of programmed cell death responsible for tissue homeostasis, in cancer cells [5-7]. The intrinsic apoptosis pathway is initiated at the mitochondria, which are permeabilized in response to intracellular death signals, releasing cytochrome c and other apoptotic factors into the cytosol and activating caspase-9 and caspase-3 [8]. The extrinsic apoptosis pathway is initiated by the binding of death receptor ligands, such as tumor necrosis factor-related apoptosis-inducing ligand (TRAIL) or CD95 ligand, to their cognate death receptors at the cell membrane [9]. Receptor trimerization leads to the intracellular recruitment of adaptor proteins, such as Fas-associated death domain (FADD), which enable the binding and activation of caspase-8 at the death-inducing signaling complex (DISC) [8]. Active caspase-8 activates caspase-3, resulting in apoptosis. As an antiapoptotic protein, cellular FLICE-inhibitory protein (cFLIP) can block death-receptor signaling by interfering with caspase-8 activation at the DISC [10].

The FADD-like interleukin-1β-converting enzyme (FLICE)-inhibitory protein cFLIP is a death effector domain (DED)-containing protein that is similar to FLICE-like inhibitory protein long (FLIPL) and FLICE-inhibitory proteins (FLIPS). These proteins play a role in apoptosis signaling, and cFLIP isoforms represent critical antiapoptotic and drug resistance factors [10]. cFLIP is highly expressed in cancer cells and TRAIL-resistant cells [11-13]. FLIP levels in TRAIL-resistant cells are regulated by the phosphatase and tensin homologue (PTEN)-AKT-mammalian target of rapamycin (mTOR) pathway, and PTEN loss and AKT activation are correlated with increased FLIPS mRNA translation, high levels of FLIPS expression, and TRAIL resistance *in vitro*, in human glioblastoma multiforme (GBM) xenografts and in primary human GBM samples [11].

Aloperine, a natural product identified in *Sophora flavescens Ait.* and other Sophora plants, such as *Sophora alopecuroides* [14-16], exhibits anti-inflammatory, antibacterial, and antiviral properties [17,18]. Clinically, aloperine has been used in China to treat patients with autoimmune diseases, such as rheumatism, lupus erythematous, and eczema [19,20]. More recently, aloperine was reported to have an antitumor effect on various cancers cells, including leukemia cell lines (HL-60, U937, and K562), esophageal cancer cells (EC109), lung cancer cells (A549), and hepatocellular carcinoma cells (HepG2) [21]. In addition, our group previously demonstrated that aloperine significantly induced apoptosis in colon cancer SW480 and HCT116 cells [22,23]. Although aloperine induced cell cycle arrest at the G2/M phase with concomitant increases in p21 and p53 and decreases in cyclin D1 and B1, its specific target(s) remain to be discovered; furthermore, it is not yet clear whether the drug can also induce apoptosis in MM cells. In this study, we demonstrate the cytotoxic effects of aloperine on primary samples and MM cell lines with or without the protective effects of bone marrow cytokines and bone marrow stromal cell (BMSC) adhesion. Importantly, we determined that aloperine functions by targeting cFLIP and phosphorylated (p)-PTEN and thus induces MM cell apoptosis through both the intrinsic and extrinsic apoptotic pathways, respectively.

## Materials and methods

### Drugs, reagents, and cell lines

Aloperine (Yanchi Dushun Biological and Chemical Co., Ltd., Ningxia, China; Batch number: 070506; purity >99%) and bortezomib (Millennium Pharmaceuticals) were dissolved in distilled water. Non-tagged TRAIL, IETD-FMK, LEHD-FMK, and Z-VAD-FMK were obtained from Bachem (Heidelberg, Germany). All other chemicals were purchased from Sigma-Aldrich (China) unless otherwise stated.

The human myeloma cell lines (OPM2, 3T3D, RPMI 8226, and NCI-H929) were kind gifts from Guangzhou Medical University and Southern Medical University. The dexamethasone-sensitive and dexamethasone-resistant cell lines MM.1S and MM1.R as well as U266 cells and the doxorubicin-resistant U266 and Dox6 cell lines were kindly provided by Sun Yat-Sen University Cancer Center. Cell lines were grown in RPMI 1640 (Gibco) with 10% fetal bovine serum (FBS) and 1% penicillin and streptomycin (100 units of penicillin and 100 μg of streptomycin).

Primary MM cells were isolated from patient bone marrow (the Second Affiliated Hospital in Guangzhou Medical University) aspirates after Ficoll-Hypaque gradient centrifugation using CD-138-positive selection and magnetic-assisted column sorting (Miltenyi) according to the manufacturer’s instructions. The purity of the MM cells was confirmed to be >90% via flow cytometric analysis using an anti-CD138 antibody (Miltenyi). CD138-negative mononuclear cells were also used to establish long-term BMSC cultures, as described previously [24]. BMSCs were obtained from MM patients and used between the third and fifth passages for all experiments. Informed written consent was obtained from all patients’ parents/guardians, and the study was approved by Guangzhou Medical University Clinical Research Ethics Committee.

To generate stable cell lines, cells were seeded in six-well tissue culture plates and transfected with cFLIPL in pBABE, cFLIPS in pCFG5-IEGZ, or the corresponding empty vectors using FuGENE 6 (Roche Applied Science, Mannheim, Germany) according to the manufacturer’s recommendations, and clones were selected using 2.5 μg/ml puromycin or 400 μg/ml Zeocin (InvivoGen, San Diego, CA, USA).

### Cell proliferation and apoptosis assays

Cell proliferation was assessed using methanethiosulfonate (MTS) assays (Promega) according to the manufacturer’s instructions. For co-culture experiments, cell proliferation was measured using the BrdUrd cell proliferation ELISA kit (Roche Diagnostics). MM cells were cultured in BMSC-coated 96-well plates for 48 h with the indicated concentrations of aloperine. Cells were pulsed with BrdUrd during the last 8 h of the 72-h culture. Apoptosis was quantified using the annexin V/propidium iodide staining assay kit (R&D Systems) according to the manufacturer’s instructions, and samples were analyzed on a FACSCalibur (BD Biosciences) flow cytometer.

### Western blotting

Western blot analyses were performed as described previously [25] using the following antibodies: caspase-8, cFLIP, caspase-9, XIAP, Bim, caspase-3, PTEN, p-PTEN (Ser380/Thr382/383), PARP, p-AKT (Ser-473), AKT, β-actin, MCL-1 (all from Cell Signaling, Beverly, MA), cIAP-2 (Epitomics, Burlingame, CA, USA), cIAP-1, survivin (both from R&D Systems), Noxa, and cFLIP (Santa Cruz Biotechnology).

### RNA interference

For transient knockdown of PTEN, cells were transfected with 150 pmol Stealth RNAi siRNA directed against PTEN, cFLIP (Invitrogen), or non-targeting control siRNA (Invitrogen) using the TransMessenger transfection reagent (Qiagen, Hilden, Germany).

### Animal models and drug treatment

SCID NOD mice (Animal Experiment Center, Southern Medical University) were used in this study. All animals were handled in strict accordance with good animal practice as defined by the relevant national and local animal welfare bodies. All animal work was approved by the Institutional Review Board of Guangzhou Medical University (permit number: GZMU (hu) 2011–0190) in accordance with the guidelines for animal use of the National Institutes of Health.

Mice were inoculated subcutaneously in the abdomen with U266 cells (5 × 10^6^ cells per mice) and randomized into the following treatment and control groups (5 per group): control (PBS), bortezomib (0.1 mg/kg, i.p., 1 dose per 3 days), TRAIL (15 mg/kg, i.p., 1 dose per 5 days), aloperine (20 mg/kg, p.o., daily), aloperine + TRAIL, and aloperine + borterzomib. Aloperine was given 24 h prior to TRAIL treatment because our goal was to induce the death receptors DR4 and/or DR5 by irradiation so that successive TRAIL treatment would enhance tumor cell apoptosis 10 days postinjection. Tumor growth was monitored every 5 days with calipers (width^2^ × length/2).

For the 5T33 myeloma mouse model (10 per group), 5 × 10^6^ 5T33 cells were injected intravenously into C57/BL KaLwRij mice (Shanghai Faculty of Life Sciences) via the tail vein. Bortezomib (0.1 mg/kg), PBS, and aloperine (20 mg/kg) treatments were initiated 7 days after injection, and blood was collected at 0, 1, 2, 3, and 4 weeks after injection.

### Measurement of the monoclonal protein IgG2b

After 1 h at room temperature, serum was obtained via centrifugation of whole blood, and samples were stored at −20°C until use. A specific analysis of mouse IgG2b in the serum was performed using a commercially available ELISA kit (IgG2b, Bethyl Laboratories).

### Bone X-rays

Following tissue dissection and fixation, bones were placed onto Kodak X-OMAT AR scientific imaging film (Kodak, Rochester, NY) and X-rayed using a Faxitron radiographic inspection unit (Model 8050–020; Field Emission Corporation, Inc., McMinnville, OR). The exposed films were developed using a RP X-OMAT processor (Model M6b; Kodak). Osteolytic lesions as small as 0.5 mm in diameter were recognized and counted as demarcated radiolucent lesions in the bone.

### Clonogenic survival assays

Colony formation assays were performed as previously described [26]. Briefly, 10,000 MM cells were seeded in 0.33% agar cultures. The colonies were fed with a medium in the presence or absence of drugs. After 18 days, the plates were imaged, and colony numbers were counted using ImageJ.

### Statistical analyses

The values presented represent the mean ± SD from at least three experiments. IC50 values for proliferation in the presence of aloperine were calculated as the concentration of drug that resulted in a 50% reduction of viable cells compared with untreated controls using MTS or BrdUrd assays. Comparisons between two groups were performed using Student’s *t* test, and three or more groups were compared using ANOVA. *P* values <0.05 or <0.001 were considered significant.

## Results

### Aloperine selectively inhibits the growth of MM cell lines and patient cells

**Figure 1:**
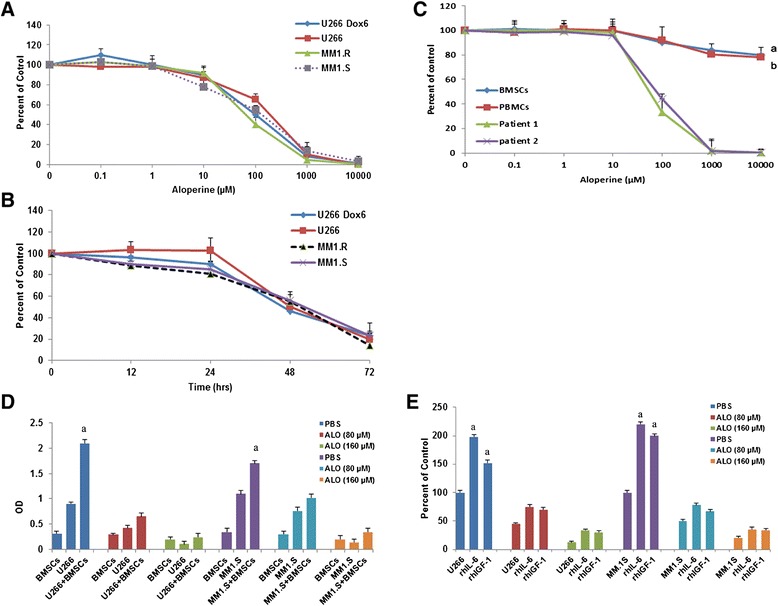
**Aloperine inhibits the growth of human MM cell lines and primary MM cells but not BMSCs or PBMCs. (A, B)** MM cell lines were treated with the indicated doses of aloperine or vehicle for 48 h **(A)** or 80 μM aloperine for 72 h **(B)**. Proliferation was measured using MTS assays, and IC50 values for different cell lines ranged from 80 to 260 μM. **(C)** MTS assays were performed on purified MM cells from 12 patients with relapsed MM whose diseases were resistant to chemotherapy, normal PBMCs, and malignant MM-derived BMSCs; two representative cases are shown. In all experiments, cells were treated for 48 h with graded doses of aloperine (^a,b^ *P* < 0.001 versus all patient MM cells). **(D)** Aloperine inhibited the proliferation of MM cells adhering to BMSCs. After BMSCs reached confluence in a 96-well plate, U266 and MM.1S cells were added and allowed to adhere for 4 h. Aloperine was added at the indicated doses, and BrdUrd proliferation assays were performed after 48 h of co-culture. BMSCs alone and MM cell lines alone served as controls (^a^ *P* < 0.001 versus U266 and MM.1S cells). **(E)** Aloperine overcomes the protective effects of recombinant human IL-6 and recombinant human IGF-I. MM.1S and U266 cells were treated for 48 h with the indicated concentrations of aloperine in the presence or absence of rhIL-6 and rhIGF-I at the indicated doses, and MTS assays were used to measure proliferation (^a^ *P* < 0.05 versus U266 and MM.1S, respectively). Data represent the means ± SD (*n* = 3). The results illustrate a representative experiment (*n* = 3).

Forty-eight hours of treatment with 0–10 mM aloperine or 72 h with 80 μM aloperine inhibited MM cell proliferation in a dose- and time-dependent manner in all cell lines tested, including dexamethasone-resistant MM.1R cells and doxorubicin-resistant U266 cells (Figure 1A, B, Additional file 1: Figure S1A, B). The IC50 of aloperine was 80–260 μM for all cell lines and primary MM cells (Figure 1C). In addition, aloperine inhibited the growth of primary patient MM cells obtained from 12 MM patients who relapsed after multiple previous therapies, including bortezomib (patients 1, 2, 3), lenalidomide (patients 4, 5, 6), and dexamethasone (patients 7, 8, 9) and melphalan (patients 10, 11, 12). Aloperine significantly inhibited patient MM cell proliferation (*P* < 0.05 for all patients; Figure 1C). As a control, aloperine did not significantly decrease the growth of BMSCs or normal PBMCs at this dose, indicating that its cytotoxicity is selective to MM cells.

### Aloperine overcomes the cytoprotective effects of MM bone marrow

Next, we utilized BrdUrd incorporation assays to investigate the cytotoxicity of aloperine on MM cells with and without adhesion to BMSCs. MM cells alone and BMSCs alone served as the controls. BrdUrd incorporation was slightly reduced in BMSCs at doses that significantly inhibited BrdUrd uptake in MM cells (Figure 1D). BrdUrd uptake by U266 cells was significantly elevated by co-culture with BMSCs (2.15-fold, *P* = 0.000), whereas a more modest upregulation was observed for MM.1S cells (1.54-fold, *P* = 0.03), suggesting that BMSCs promote MM cell proliferation. Similar results were observed in other MM cell lines, including RPM1-8226 and OPM2 (Additional file 1: Figure S1C). However, aloperine prevented cell proliferation in MM-BMSC co-cultures (*P* = 0.06 and *P* = 0.052 for U266 and MM.1S, respectively; Figure 1D). These data suggest that aloperine overcomes the cytoprotective effects of the MM-host BM microenvironment.

### Aloperine reduces the cytoprotective effects of IL-6 and IGF-I on MM cells

Because previous reports demonstrated that IL-6 and IGF-I secretion from BMSCs confers growth, survival, and drug resistance in MM cells [27], we next examined whether aloperine retains its ability to trigger MM cell death in the presence of these cytokines.

U266 and MM.1S cells were treated with different doses of aloperine for 48 h. Neither cytokine protected MM cells from aloperine-mediated apoptosis (Figure 1E); however, both cytokines promoted MM cell growth (Figure 1E). The aloperine-mediated inhibition of MM cell proliferation was unchanged in the presence of these cytokines (*P* = 0.073 and 0.084 for U266 and MM.1S, respectively; Figure 1E). Similar results were observed in other MM cell lines (*P* = 0.101 and 0.064 for RPM1 and OPM2, respectively; Additional file 1: Figure S1D).

### Aloperine triggers dose-dependent, caspase-mediated apoptosis

**Figure 2:**
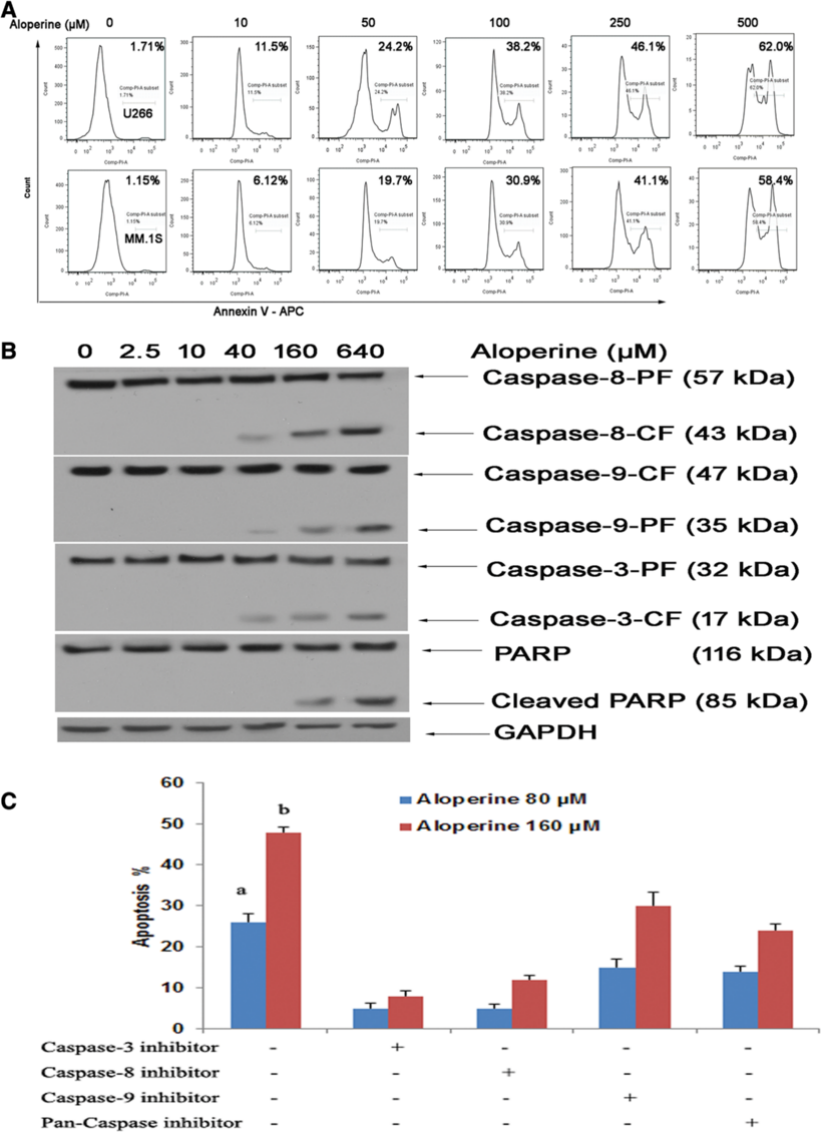
**Aloperine induces cell apoptosis in a dose-dependent manner. (A)** U266 and MM.1S cells were treated with various concentrations of aloperine for 48 h, harvested, and analyzed by flow cytometry. **(B)** Aloperine activates caspases. Western blot analysis of lysates from U266 and MM.1S cells treated with 80 μM aloperine for the indicated durations with or without anti-caspase-8 or anti-caspase-9 antibodies. PF, full length; CF, cleaved fragment. Blots are representative of three independent experiments. β-Actin was assessed by Western blotting. All proteins levels were quantified densitometrically and are normalized to β-actin. **(C)** U266 cells were treated with 80 or 160 μM aloperine and the indicated agents for 48 h (as described in panel **(B)**) in the presence or absence of biochemical inhibitors of caspase-3, caspase-8, or caspase-9, and apoptosis was assessed using annexin V/propidium iodide staining. Data represent the mean ± SD. The results are from a representative experiment (*n* = 3) (^a,b^ *P* < 0.001 versus no inhibitors in MM cells).

Treatment of U266 and MM.1S cells with aloperine induced apoptosis in a dose-dependent manner (Figure 2A). Next, we analyzed whether treatment with aloperine activated caspases, which are key executioners of apoptosis. To verify the rapid caspase activation observed using the FACSscan assay, we monitored the effects of aloperine on different caspases using Western blot analysis. We observed an obvious accumulation of cleaved caspase-8 and, to a lesser extent, cleaved caspase-9 and caspase-3 (Figure 2B). Indeed, the inhibition of caspase-8 using the specific pharmacological inhibitor IETD-FMK significantly decreased aloperine-induced cell apoptosis (*P* < 0.05, versus control), whereas the inhibition of caspase-9 (LEHD-FMK) moderately blocked aloperine-triggered U266 cell death (*P* < 0.05) (Figure 2C). Moreover, incubation of U266 cells with a pan-caspase inhibitor (Z-VAD-FMK) markedly abrogated aloperine-induced apoptosis (Figure 2C).

### Aloperine activates caspase-8 by downregulating cFLIP

**Figure 3:**
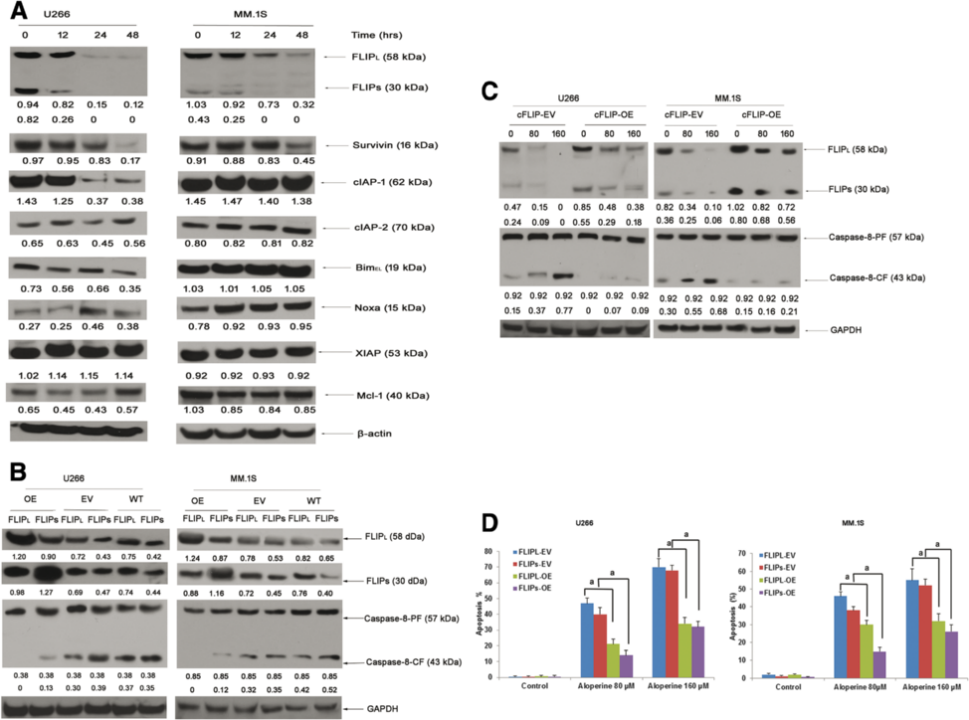
**Aloperine activates caspase-8 via downregulation of cFLIP. (A)** U266 and MM.1S cells were treated with 80 μM aloperine for the indicated durations. Protein expression levels of cFLIP, Noxa, Bim_el_, cIAP-1, cIAP-2, XIAP, survivin, Mcl-1, and β-actin were analyzed using Western blotting after treatment for 0, 12, 24, or 48 h. **(B,** **C)** U266 cells were transfected with FLIPS, FLIPL, or the corresponding empty vectors (empty vector FLIPL: FLIPL-EV; empty vector FLIPS: FLIPS-EV; FLIPL and FLIPS overexpression: FLIPL-OE and FLIPS-OE; WT: FLIPL, FLIPS). Expression of cFLIP and caspase-8 was verified by Western blotting in EV and cFLIP OE U266 cells without **(B)** or with **(C)** different doses of aloperine. **(D)** Cells were treated with 80 or 160 μM aloperine for 48 h, and apoptosis was determined via FACS analysis of DNA fragmentation of propidium iodide-stained nuclei. Background apoptosis: FLIPL-EV: 1.5%; FLIPS-EV: 1.4%; FLIPL: 0.9%; FLIPS: 1.3%. Data represent the mean ± SD of three independent experiments carried out in triplicate; ^a^ *P* < 0.05. GAPDH/β-actin was assessed by Western blotting. All proteins levels were quantified densitometrically and normalized to GAPDH/β-actin.

Previous studies have established an antiapoptotic role for cFLIP [28]. Our data indicate that aloperine decreased both FLIPL and FLIPS in a time-dependent manner (Figure 3A). In addition, aloperine slightly upregulated Noxa in MM.1S cells; however, this effect was not observed in U266 cells, and changes in the expression of BimEL and cIAP-1 were not consistent in both cell lines (Figure 3A). Furthermore, levels of clAP-2, XIAP, and MCL-1 were unchanged in both cell lines. In contrast, survivin was reduced 48 h after aloperine treatment in U266 cells (Figure 3A), indicating that survivin is likely a key mediator of this sensitization. Importantly, FLIPL and FLIPS levels were decreased in aloperine-pretreated cells, coinciding with an increase in caspase-8 cleavage fragments (Figure 2B).

To determine whether caspase-8 mediates aloperine-induced apoptosis through cFLIP, we overexpressed both isoforms of cFLIP (Figure 3B). Indeed, overexpression of FLIPL or FLIPS blocked caspase-8 cleavage and significantly decreased aloperine-induced apoptosis (Figure 3C, D). To further test the functional relevance of the aloperine-mediated downregulation of cFLIP, we silenced cFLIP expression by RNA interference. Importantly, knockdown of cFLIP significantly increased aloperine-induced apoptosis (Additional file 1: Figure S2).

### Aloperine activates caspase-9 by downregulating phosphorylated PTEN

**Figure 4:**
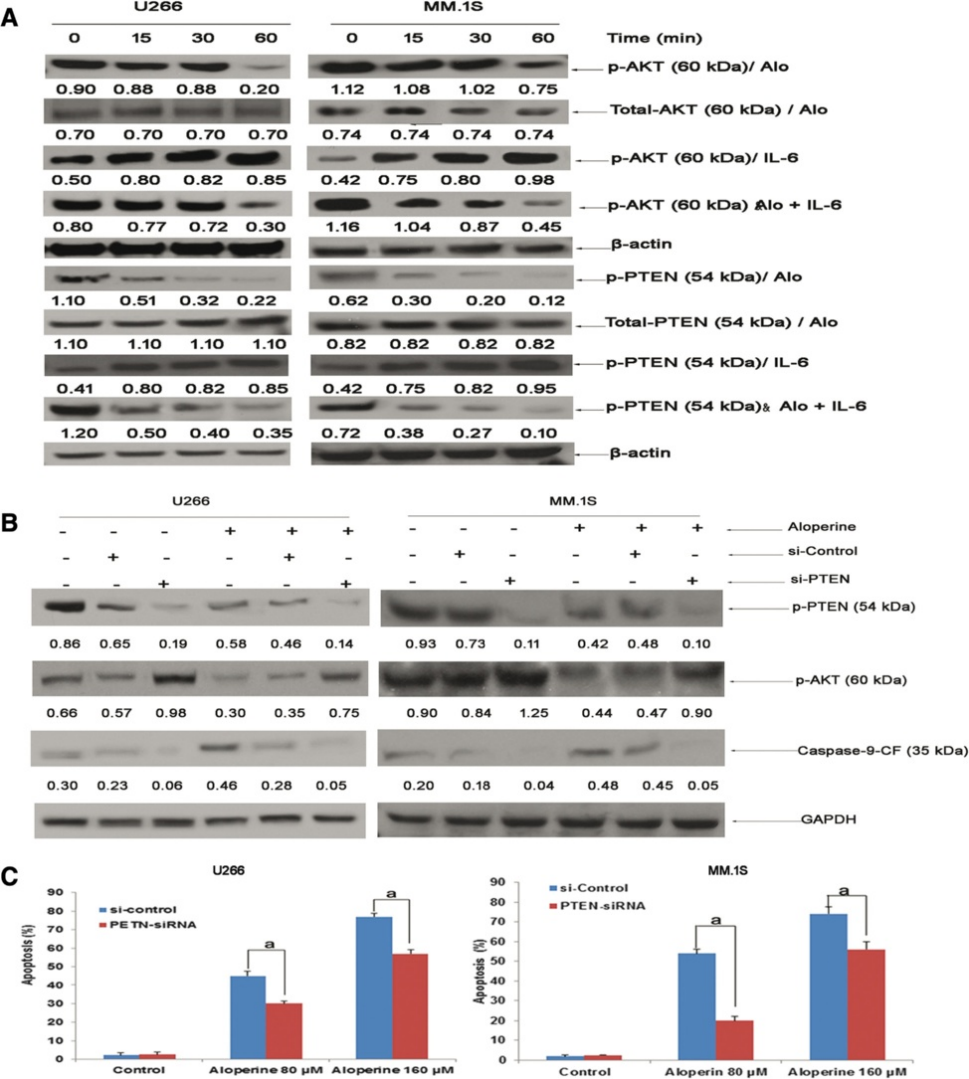
**Aloperine activates caspase-9 via downregulation of p-PTEN. (A)** U266 and MM.1S cells were treated with aloperine (80 μM), IL-6 (10 ng/ml), or both for the indicated times. The expression levels of p-PTEN and p-AKT were determined using Western blotting. **(B)** U266 cells were transiently transfected with siRNA against PTEN or non-targeting control siRNA and then treated with 80 μM aloperine for 48 h prior to evaluating the expression of caspase-9, p-PTEN, p-AKT, and β-actin by Western blotting. **(C)** Transfected cells were treated with 80 or 160 μM aloperine for 48 h, and apoptosis was determined via FACS analysis of DNA fragmentation of propidium iodide-stained nuclei. Background apoptosis: si-control, 2.3%; PTEN siRNA, 2.6%. Data represent the mean ± SD of three independent experiments carried out in triplicate; ^a^ *P* < 0.001. GAPDH was assessed by Western blotting. All protein levels were quantified densitometrically and normalized to GAPDH.

The PTEN-AKT pathway plays an important role in cancer, and loss of PTEN function increases p-AKT levels, promoting the accumulation of caspase-9 [29]. Thus, we sought to determine whether increased PTEN in aloperine-treated cancer cells reduced the levels of p-AKT and activated caspase-9. Figure 4A demonstrates that aloperine induced AKT and PTEN phosphorylation. The maximum decrease in p-AKT was observed at 60 min, whereas p-PTEN peaked at 15 min. The increase in p-PTEN thus occurred earlier than that of p-AKT, indicating that PTEN plays a key role in the aloperine-mediated activation of PTEN-AKT. Compared with caspase-9, there was less change in total PTEN and AKT protein expression in MM cells exposed to aloperine for 24 or 48 h (data not shown). Moreover, increased p-AKT and p-PTEN were observed in a time-dependent manner in IL-6-treated MM cells, but IL-6 did not interfere with the aloperine-mediated decreases in p-AKT and p-PTEN, indicating that aloperine reduces the cytoprotective effects of IL-6 on MM cells (Figure 4A).

To further demonstrate that PTEN plays a key role in aloperine-mediated cytotoxicity in MM cells, siRNA against PTEN was transfected into U226 and MM.1S cells. PTEN knockdown blocked the ability of aloperine to reduce p-AKT and increase caspase-9 protein in U266 cells, and aloperine-mediated apoptosis was also inhibited compared with si-control U266 cells (Figure 4B, C). These results suggest that blockade of the PTEN-AKT-caspase-9 signaling pathway decreases the effect of aloperine in cancer cells, suggesting that aloperine-induced apoptosis is at least partly mediated by activated caspase-9.

### Aloperine-mediated inhibition of clonogenic survival is irreversible and associated with a decrease in FLIPL or FLIPS and p-PTEN

**Figure 5:**
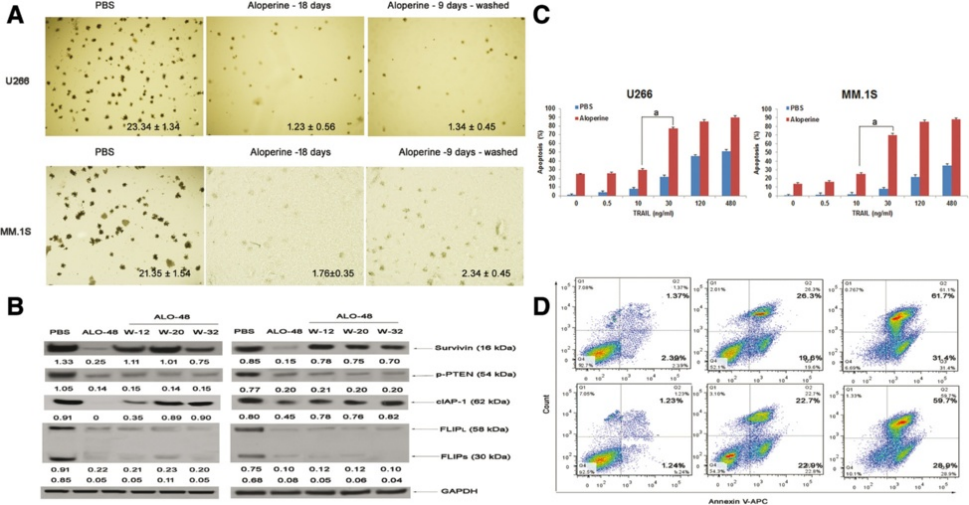
**Aloperine-mediated inhibition of clonogenic survival is irreversible. (A)** Cells were treated with aloperine (ALO) for 9 days to inhibit clonogenic survival. Cells were then washed and cultured in a drug-free medium for an additional 9 days (ALO-9 days-washed) or continuously cultured in a drug-containing medium for an additional 9 days (ALO-18 days), followed by colony staining. **(B)** Cells were treated by aloperine for 48 h, washed at the indicated times and harvested for Western blotting analysis. **(C)** Aloperine displays a synergistic effect with TRAIL. U266 (left on panel) and MM.1S (right on panel) cells were treated for 48 h with (red bars) or without (blue bars) aloperine, followed by the addition of the indicated concentrations of TRAIL for 48 h. Apoptosis was determined via FACS analysis of DNA fragmentation of propidium iodide-stained nuclei. Data represent the mean ± SD of at least three independent experiments carried out at least in duplicate; ^a^ *P* < 0.001. GAPDH was assessed by Western blotting. All protein levels were quantified densitometrically and normalized to GAPDH. **(D)** U266 and MM.1S cells were treated with 80 μM aloperine and aloperine (80 μM) + borterzomib (2.5 nM) for 48 h, harvested, and analyzed by flow cytometry.

Aloperine inhibited clonogenic survival in U266 and MM.1S cells in a dose-dependent (Additional file 1: Figure S3) and irreversible manner. Cells were treated with aloperine (8 μM) for 9 days to inhibit clonogenic formation, followed by either drug removal and cell culture in a drug-free medium for an additional 9 days or incubation in a drug-containing medium for an additional 9 days. Aloperine-mediated suppression of clonal survival in U266 (Figure 5A, left on panel) and MM.1S cells (Figure 5A, right on panel) was not reversible after the removal of the drug.

As shown in Figure 5B, the cancer cell apoptotic factors, survivin and cALP-1, were decreased 48 h after aloperine treatment and increased upon drug removal, suggesting that drug removal reactivated an antiapoptotic mechanism.

Interestingly, FLIPL and FLIPS levels remained low in U266 cells (Figure 5B) and continued to decline in MM.1S cells (data not shown) after the removal of aloperine. Moreover, p-PTEN also remained at low levels after drug removal. These results suggest that persistent FLIPL or FLIPS degradation and sustained activation of the PTEN-AKT signaling pathway after drug removal contribute to the irreversibility of aloperine-mediated inhibition of clonogenic formation.

### Aloperine augments the growth inhibition of MM cells treated with TRAIL or bortezomib

TRAIL interaction with DR4 and DR5 transduces the death receptor (extrinsic) and mitochondrial apoptosis signaling pathways through the activation of caspase-8 and caspase-10; therefore, TRAIL is usually regarded as a drug that treats cancer cells through cFLIP [30,31]. To investigate whether aloperine is a feasible agent to enhance TRAIL sensitivity in MM, we analyzed the cytotoxicity of aloperine in combination with TRAIL in MM cell lines. Figure 5C, D demonstrates that aloperine strongly increases TRAIL-induced apoptosis in different cell lines in a dose-dependent manner compared with treatment with either drug alone (*P* < 0.05) for TRAIL concentrations up to 30 ng/ml.

Bortezomib is by far one of most common treatments for MM patients in the clinic. Borterzomib was regarded as a positive control for our experiments; however, it is unknown that aloperine induced MM apoptosis with borterzomib. Figure 5C, D showed that aloperine + bortezomib induced apoptosis more than aloperine only.

### Aloperine suppresses human MM cell growth *in vivo*

**Figure 6:**
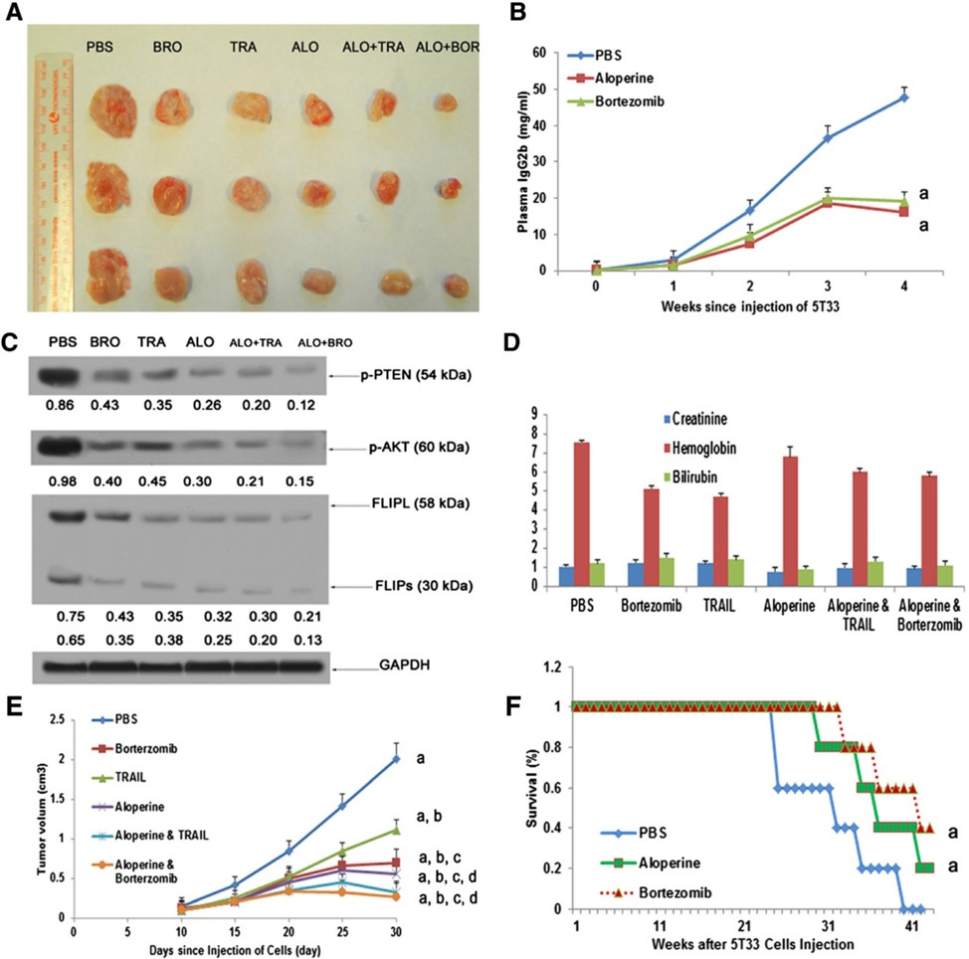
**Aloperine suppresses human MM cell growth** ***in vivo*** **. (A)** The mean ± SD of tumor volume (mm^3^) of each treatment group (*n* = 5/group) versus time (days). Mice were treated with PBS, TRAIL, aloperine, aloperine + TRAIL/bortezomib at the indicated doses for 30 days. **(B)** A significant delay in tumor growth was observed in the treated mice compared with PBS-treated control mice (^a^ *P* < 0.001 versus PBS). Bars represent the mean ± SD. **(C)** Tumor lysates from control and drug-treated mice were subjected to Western blot analysis using anti-cFLIP, p-AKT, AKT, and p-PTEN antibodies; ALO, aloperine; Bor, bortezomib. **(D)** Mice were treated with the indicated drugs for 30 days, and blood samples were obtained to measure serum bilirubin, hemoglobin, and creatine levels using the Quantichrom Creatinine, Bilirubin, and Hemoglobin Assay kit (BioAssay Systems). Aloperine cured 5T33 multiple myeloma mice. 5T33 MM mice were treated with aloperine and bortezomib for 40–45 days. **E**: IgG2b expression in aloperine- and bortezomib-treated mice was measured using ELISA assays. ^a^ *P* < 0.001 versus PBS; ^b^ *P* < 0.001 versus bortezomib; ^c^ *P* < 0.05 versus TRAIL; ^d^ *P* < 0.05 versus aloperine. **(F)** Kaplan-Meier plots of mice treated with the indicated concentrations of PBS, aloperine, or bortezomib. Survival was significantly increased in aloperine- and bortezomib-treated mice compared with the untreated group (PBS). ^a^ *P* < 0.05 for the PBS group. ^a^ *P* < 0.001 versus the PBS group. Values represent the mean ± SD.

Because sequential treatment of U266 cells with aloperine followed by TRAIL enhanced apoptosis in a synergistic manner *in vitro*, we sought to validate this treatment combination *in vivo*. According to our *in vitro* data, a synergistic effect of TRAIL and aloperine was observed when TRAIL was added 24 h before aloperine. Therefore, after tumor formation, mice were exposed to TRAIL, bortezomib, aloperine, aloperine + TRAIL, or aloperine + bortezomib, as described in the ‘Materials and methods.’ Treatment of mice with bortezomib, aloperine, TRAIL, aloperine + TRAIL, or aloperine + bortezomib inhibited tumor growth (*P* < 0.05) (Figure 6A, B). Interestingly, tumor growth was inhibited to a greater extent in response to aloperine followed by TRAIL or bortezomib administration or aloperine alone compared with TRAIL alone (*P* < 0.05). Importantly, the inhibition of tumor growth in the aloperine + TRAIL or aloperine + bortezomib groups was significantly superior to that of aloperine alone (*P* < 0.05), indicating that aloperine augments the growth inhibition of MM cells treated with TRAIL or bortezomib *in vivo*. Results consistent with the inhibition of tumor growth were observed at the protein level by Western blotting (Figure 6C). Toxicity was not observed in the liver tissues of treated mice, as determined by H&E staining (Additional file 1: Figure S4). Moreover, aloperine did not trigger leucopenia/neutropenia, thrombocytopenia, or hepatic damage (Figure 6D). These findings suggest that aloperine markedly reduces tumor growth and is well tolerated *in vivo*.

### Aloperine reduces IgG2b and bone lesions in 5T33 mice harboring myeloma cells and increases survival

Aloperine-mediated the inhibition of MM cell growth was repeated in 5T33 MM mice. We observed a time-dependent increase in serum monoclonal IgG2b protein levels in fresh marrow cells from the femurs of 5T33 MM mice. Interesting, the level of IgG2b was significantly decreased by treatment with aloperine compared with PBS (*P* < 0.05, Figure 6E). In addition, compared with PBS, multiple lytic lesions and diffuse osteopenia were obviously reduced in the aloperine- and bortezomib-treated groups, as determined via X-ray (Additional file 1: Figure S5). Survival of the mice in the aloperine- and bortezomib-treated groups was higher than that in the PBS group (Figure 6F, *P* < 0.05 and *P* < 0.05, respectively).

## Discussion

In this study, we demonstrated that aloperine decreases MM cell viability without affecting normal lymphocyte viability, suggesting that it is a selective cytotoxic agent against MM cells compared with normal BMSCs and PBMCs. Furthermore, to explain why aloperine selectively treated MM cells, malignant MM-derived BMSCs were added to the experiment. The bone marrow microenvironment promotes MM proliferation and growth via the secretion of IL-6. IL-6 is a major growth factor and antiapoptotic cytokine for MM cells [32]; the importance of IL-6 as an autocrine growth factor for MM cells is widely accepted [24]. In the current experiment, aloperine overcame the proliferative effects of IL-6, IGF-I, and BMSCs, suggesting that aloperine could be efficacious in treating MM.

Genetic heterogeneity and drug resistance are hallmarks of MM [33,34] that explain, at least in part, differences in the cytotoxic activity of different agents. Importantly, our data demonstrate that aloperine displays anti-MM activity in a panel of MM cell lines, including sensitive and resistant cell lines, such as U266, U266 Dox6, MM.1S, and MM1.R, as well as cytogenetically distinct MM cells. We observed similar responses in patient-derived MM cells that were resistant to anti-MM therapies such as bortezomib, lenalidomide, dexamethasone, or melphalan. These data indicate that aloperine presents a special characteristic that sets it apart from general chemotherapy drugs for MM.

Mechanistic studies indicated that the anti-MM activity of aloperine is associated with the activation of caspases, cFLIP, Bim, and Noxa, among others. Notably, aloperine triggers robust caspase-8 cleavage; however, modest caspase-9 cleavage was also observed. These data are consistent with our findings that biochemical inhibitors of caspase-8 partly abrogate the anti-MM activity of aloperine, suggesting that caspase-9 compensates to induce MM apoptosis. Because cFLIP is structurally similar to caspase-8 and the expression of cFLIP isoforms is increased in various types of cancer, including MM, cFLIP represents a critical target for therapeutic intervention, namely via inhibiting its transcription and posttranscriptional modifications [35]. In this study, we demonstrate that aloperine significantly decreases the expression of cFLIP and activates procaspase-8 in MM cells.

Furthermore, aloperine induced MM cell apoptosis in MM cells overexpressing cFLIP, suggesting that cFLIP downregulation is important for aloperine cytotoxicity. Moreover, cFLIP overexpression conferred TRAIL resistance; therefore, the aloperine-mediated sensitization to TRAIL observed in our study is secondary to cFLIP downregulation. TRAIL is the leading death receptor ligand in clinical development with selective activity against cancer cells [36]. However, drug resistance severely hinders its efficacy. Our data suggest that aloperine may not only help overcome cFLIP-mediated TRAIL resistance but could also enhance the ability of TRAIL to inhibit MM cell growth.

PTEN loss causes an mTOR-dependent increase in the translation of FLIPS mRNA, increases the levels of antiapoptotic FLIP proteins, and increases TRAIL resistance [11]. PTEN suppresses AKT phosphorylation and activates caspase-9 to induce apoptosis in MM cells [29]. Furthermore, enhanced phosphorylation of PTEN at the Ser380/Thr382/Thr383 cluster was recently shown to inactivate PTEN and enhance PI3K-AKT pathway activation in adult T-cell leukemia-lymphoma (ATLL) and other cancers [37]. Our study is the first to show that aloperine regulates AKT and PTEN phosphorylation. Declines in AKT and PTEN phosphorylation occurred in a time-dependent manner when MM cells were treated by aloperine. The decrease in p-PTEN occurred significantly earlier than that in p-AKT and resulted in elevated levels of p-AKT, thus further demonstrating that PTEN plays a key role in the PTEN-AKT pathway. Importantly, knockdown of PTEN partially rescued cells from aloperine-mediated upregulation of caspase-9 and significantly reduced aloperine-induced apoptosis (Figure 4C). These data suggest that aloperine kills cancer cells via the PTEN-AKT-caspase-9 signaling pathway.

At low drug concentrations, the ability of aloperine to inhibit clonogenic survival was irreversible. Moreover, the ability of aloperine to inhibit MM survival was largely dependent on cFLIP and the PTEN-AKT pathway. Therefore, it is possible to use low doses of aloperine to achieve a greater therapeutic index because normal fibroblasts are much more resistant to the drug than MM cells are.

In addition to our *in vitro* studies, we also examined the anti-MM activity of aloperine *in vivo* using a human MM xenograft mouse model. To demonstrate drug activity, bortezomib and TRAIL were included as positive controls. Marked tumor growth inhibition was observed following treatment with bortezomib, TRAIL, aloperine, or aloperine + TRAIL/bortezomib compared with treatment with PBS alone. Interestingly, the combination of aloperine and TRAIL/borterzomib showed a synergistic inhibitory effect on MM growth compared with TRAIL or aloperine alone.

5T33 models have been considered as suitable models for human MM [38]. This murine model of human myeloma bone disease includes radiologic evidence of osteolysis, hypercalcemia, and increased osteoclastic bone resorption associated with monoclonal gammopathy [39]. This animal model of human myeloma bone disease can be applied to determine the efficacy of potential therapeutic agents. The 5T33 model that we utilized is a systemic model of aggressive MM with the unambiguous endpoint of hind limb paralysis [40]. In our study, aloperine not only reduced the expression of IgG2b and bone lesions in 5T33 mice but also increased survival. Importantly, aloperine treatment was not toxic, as no differences in creatinine, hemoglobin, or bilirubin were noted. Therefore, these findings demonstrate that aloperine elicits a dual effect in MM tumors, increases apoptosis, and decreases proliferation.

## Additional file

Additional file 1:
**Supplemental Materials. Figure S1.** Aloperine treatment. **Figure S2.** Knockdown of cFLIP sensitizes TRAIL-induced apoptosis. **Figure S3.** Aloperine inhibited clonogenic survival. **Figure S4.** Images of dislodged tumors and HE staining did not detect any toxic response in liver. **Figure S5.** Bone damage in three groups was measured by x-ray.
