# Supplementary material for: Aloperine executes antitumor effects against multiple myeloma through dual apoptotic mechanisms
Source: J Hematol Oncol. 2015 Mar 15;8:26. doi: 10.1186/s13045-015-0120-x (PMC4377192; doi:10.1186/s13045-015-0120-x)
Supplement: Additional file 1: — Supplemental Materials. Figure S1. Aloperine treatment. Figure S2. Knockdown of cFLIP sensitizes TRAIL-induced apoptosis. Figure S3. Aloperine inhibited clonogenic survival. Figure S4. Images of dislodged tumors and HE staining did not detect any toxic response in liver. Figure S5. Bone damage in three groups was measured by x-ray. [file 13045_2015_120_MOESM1_ESM.docx]

**Supplemental Materials**


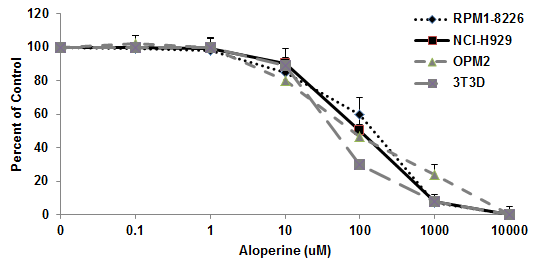


Fig. 1A


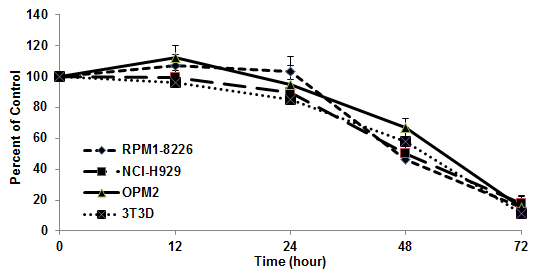


Fig. 1B


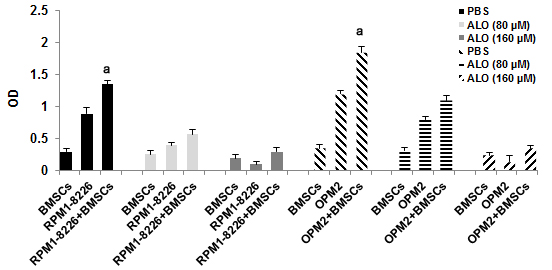


Fig. 1C


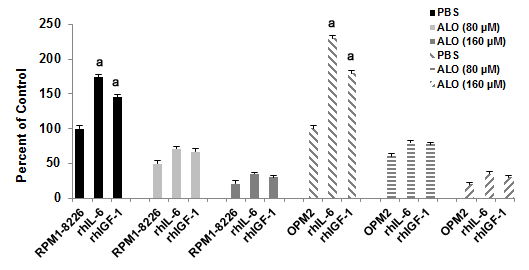


Fig. 1D

Fig.S1. A broad array of other MM cell lines (RPM1-8226, NCI-H929, OPM2, and 3T3D) were treated with the indicated doses of aloperine or vehicle for 48 hrs (**A**) or with 80 uM aloperine for 72 hrs (**B**). **C**, Aloperine inhibits the proliferation of MM cells adhering to BMSCs. After BMSCs reached confluence in a 96-well plate, RMP1-8226 and OPM2 cells were added and allowed to adhere for 4 hrs. Aloperine was added at the indicated doses, and BrdUrd proliferation assays were performed after 48 hrs of co-culture. BMSCs alone and MM cell lines alone served as controls (a, P<0.001 versus RMP1-8226 and OPM2 cells, respectively). **D**, Aloperine treatment overcomes the protective effect of recombinant human IL-6 and recombinant human IGF-I. RMP1-8226 and OPM2 cells were treated for 48 hrs with the indicated concentrations of aloperine in the presence or absence of rhIL-6 and rhIGF-I at the indicated doses, respectively, and then analyzed for proliferation by an MTS assay (a, P<0.001 versus RMP1-8226 and OPM2, respectively). The data shown are the means ±SD (n = 3). The results are from a representative experiment (n = 3).


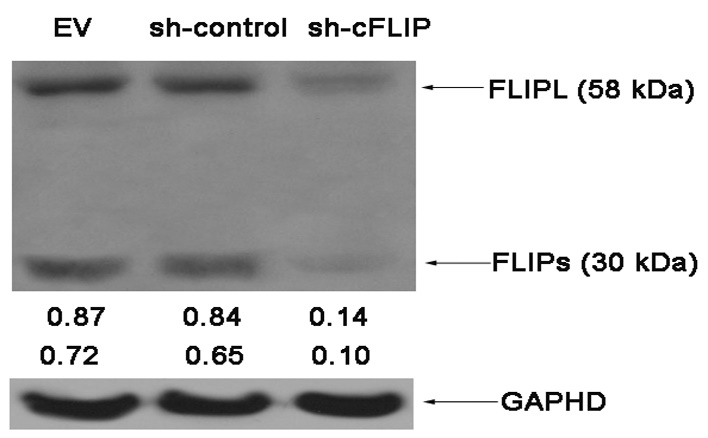


Fig. 2A


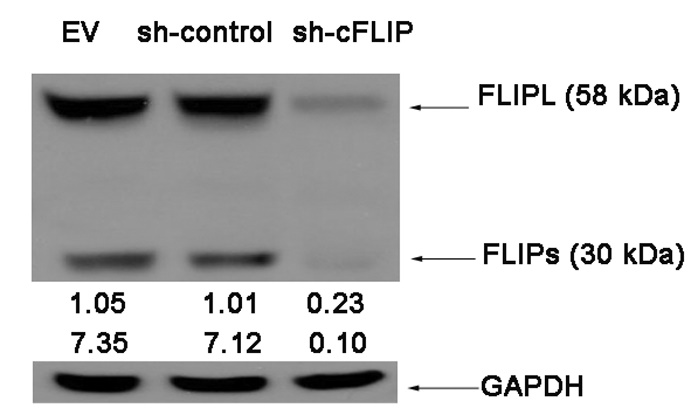


Fig. 2B


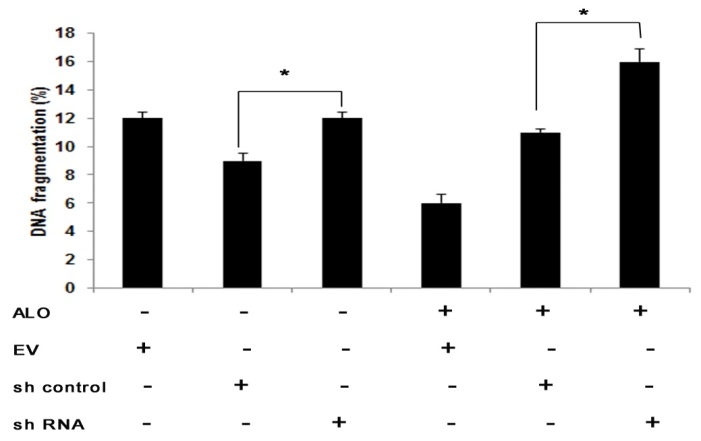


Fig. 2C


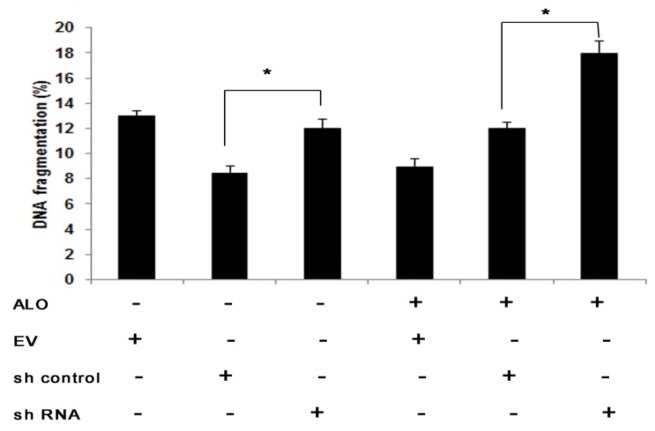


Fig. 2D

Fig. S2. Knockdown of cFLIP sensitizes TRAIL-induced apoptosis. U266 cells (A) and MM.1S (B) were stably transduced with empty vector (EV), control shRNA or two distinct cFLIP shRNA vectors. Protein expression of cFLIP was assessed by Western blotting. GAPDH served as the loading control. U266 cells (C) and MM.1S (D) stably transduced with empty vector (EV), control shRNA or cFLIP shRNA vectors were treated for 24 h with 80 µM aloperine followed by the addition of the indicated concentrations of TRAIL for 24 h. Apoptosis was determined by FACS analysis of DNA fragmentation of propidium iodide-stained nuclei. The mean + SD of three independent experiments carried out in triplicate are shown; * P < 0.05.


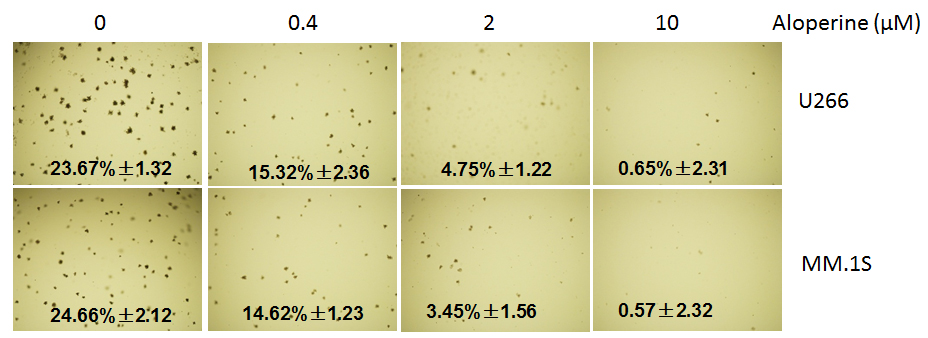


Fig. 3

#### Fig. S3. Aloperine inhibited clonogenic survival. Cells were treated with different doses of aloperine for 18 days to inhibit clonogenic survival.


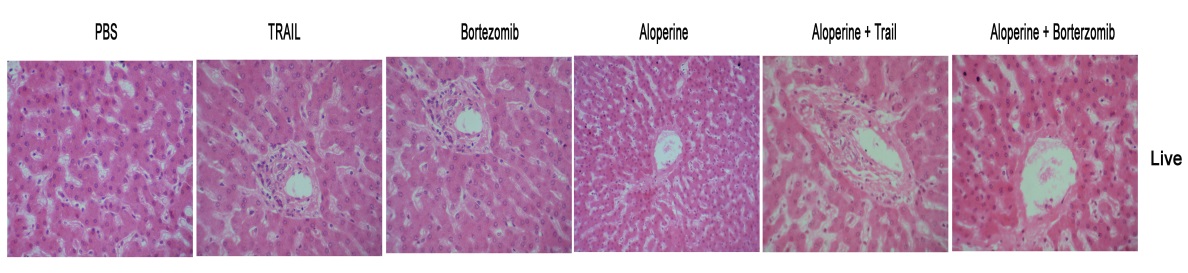


#### Fig.S4, Images of dislodged tumors and HE staining did not detect any toxic response in liver.


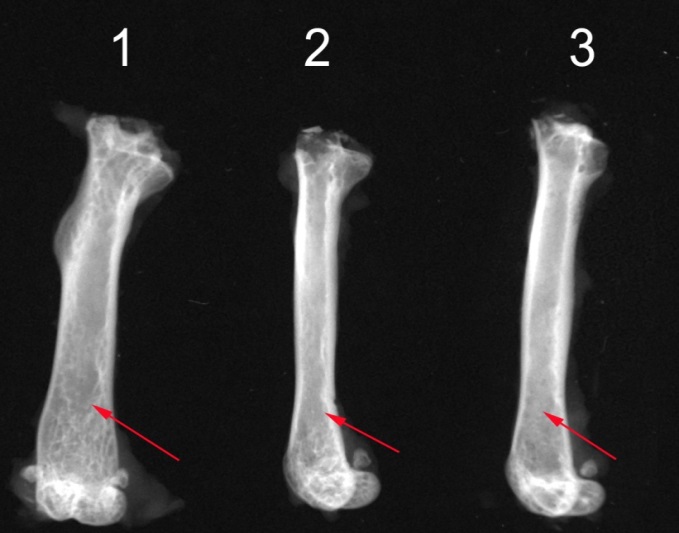


Fig. S5. Bone damage in three groups was measured by x-ray. (1) PBS group; (2) 0.1 mg/kg bortezomib; (3) 20 mg/kg aloperine. The red arrows indicate bone damage.
